# Early life stress induces age-dependent epigenetic changes in p11 gene expression in male mice

**DOI:** 10.1038/s41598-021-89593-7

**Published:** 2021-09-01

**Authors:** Mi Kyoung Seo, Jung Goo Lee, Sung Woo Park

**Affiliations:** 1 Paik Institute for Clinical Research, Inje University, Busan, 47392 Republic of Korea; 2 Department of Psychiatry, College of Medicine, Haeundae Paik Hospital, Inje University, Busan, 48108 Republic of Korea; 3 Department of Convergence Biomedical Science, College of Medicine, Inje University, Busan, 47392 Republic of Korea

**Keywords:** Genetics, Neuroscience

## Abstract

Early life stress (ELS) causes long-lasting changes in gene expression through epigenetic mechanisms. However, little is known about the effects of ELS in adulthood, specifically across different age groups. In this study, the epigenetic modifications of p11 expression in adult mice subjected to ELS were investigated in different stages of adulthood. Pups experienced maternal separation (MS) for 3 h daily from postnatal day 1 to 21. At young and middle adulthood, behavioral test, hippocampal p11 expression levels, and levels of histone acetylation and methylation and DNA methylation at the hippocampal p11 promoter were measured. Middle-aged, but not young adult, MS mice exhibited increased immobility time in the forced swimming test. Concurrent with reduced hippocampal p11 levels, mice in both age groups showed a decrease in histone acetylation (AcH3) and permissive histone methylation (H3K4me3) at the p11 promoter, as well as an increase in repressive histone methylation (H3K27me3). Moreover, our results showed that the expression, AcH3 and H3Kme3 levels of p11 gene in response to MS were reduced with age. DNA methylation analysis of the p11 promoter revealed increased CpG methylation in middle-aged MS mice only. The results highlight the age-dependent deleterious effects of ELS on the epigenetic modifications of p11 transcription.

## Introduction

Children exposed to early life stress (ELS) such as neglect and abuse have a significantly increased risk of developing depression^1,2^. In human and animal studies, ELS has been reported to induce a depression-like phenotype in adulthood^3,4^. These studies are focused on the epigenetic mechanisms, for example, DNA methylation and histone modification, by which ELS may alter the expression of genes involved in the stress response, including brain-derived neurotrophic factor (BDNF), glucocorticoid receptor (GR; *NR3C1*), and corticotrophin-releasing factor^3,4^. The emphasis of these papers is mainly on the detrimental effects of ELS. However, little is known regarding the effects of ELS on behaviors and epigenetic mechanisms over the lifespan, especially among different adult age groups.

DNA methylation and histone modification, two representative epigenetic mechanisms, control gene transcription by affecting chromatin remodeling^5^. DNA methylation at the 5′-C-phosphate-G-3′ (CpG) dinucleotide is classically associated with transcriptional repression^6^. Regions with a high density of CpGs are known as CpG islands; they are often found in the gene regulatory regions of promoters. Methylated CpGs in a promoter can induce gene silencing by blocking transcription factor binding or by attracting proteins that cause chromatin remodeling^6^. Histone modifications affect chromatin structure via post-translational modification, for example, acetylation and methylation, of the lysine (K) residues in the histone tails^7^. Acetylation of histone H3 or H4 relaxes the interaction between DNA and histone, allowing the transcriptional machinery access to the promoter, thereby activating transcription^7^. K4 and K19 on histone H3 are commonly modified in gene transcription activation^8^. Histone methylation, in contrast, is associated with both gene activation (H3K4 and H3K36) and repression (H3K9, H3K27, and H4K20), depending on the K residue methylated and its valence state (i.e., mono-, di-, or tri-methylation)^9^.

In animals that had experienced ELS, those in young adulthood exhibit decreased H3K9 di-methylation (me2) at the BNDF IV promoter and, accordingly, increased BDNF IV expression, whereas those in middle adulthood show increased repressive H3K9me2 at the BDNF IV promoter and concurrent decreased BDNF IV expression^10^. In a rodent model, caregiver's infant maltreatment increases the DNA methylation of BDNF through the lifespan to adulthood, consistent with a decrease in BDNF expression in the adult hippocampus^11^. Exposure to caregiver maltreatment also alters the level of expression of genes important in regulating DNA methylation patterns (*Dnmt1, Dnmt3a, MeCP2, Gadd45b and Hdac1*) in the adult medial prefrontal cortex^12^.

Additionally, ELS was found to be associated with an age-dependent decline in cognition. In another study, ELS was demonstrated to be associated with long-term deleterious effects on the regulation of GR expression via histone acetylation and methylation as well as on depression-like behavior^13^. The effects of ELS on the epigenetic regulation of other stress-related genes besides BDNF and GR are less well-known and warrant further investigation.

P11 (*S100A10*) plays an important role in depression and antidepressant action^14^. In a study using yeast two-hybrid screening, p11 was initially identified as a binding protein to the serotonin 1B (5-HT_1B_) receptor; the 5-HT_1B_ receptor regulates serotonin neurotransmission^15^. p11 interacts with the 5-HT_1B_ receptor by increasing its trafficking to the cell surface, where it binds to serotonin released from presynaptic neurons. Consequently, 5-HT_1B_ receptor signaling efficacy is enhanced. Patients with depression show reduced p11 levels in the brain, and p11 knockout mice display a depression-like phenotype^16–18^. Antidepressant therapy, including selective serotonin reuptake inhibitors, enhances p11 expression^15,19,20^. Furthermore, p11 gene transfer therapy effectively reverses depression-like behavior in mice^17^. In addition, p11 is critical in the antidepressant actions of BDNF^20^. However, whether epigenetic regulation of the p11 gene following ELS is altered across the life span is unknown. Maternal separation (MS) is widely used as a model to examine the effects of ELS^21^. Our previous study reported that the decrease of hippocampal BDNF expression induced by MS was associated with decreased histone H3 acetylation at the BDNF exon I promoter^22^. Previous study has only been investigated in young adulthood. There is still relatively little information on age-related changes in epigenetic regulation from young adult to middle ages. According to meta-analytic study using 1739 C57BL/6J wild-type mice, age-related changes from young adulthood to middle age (2–12 months old) occurred in a variety of behaviors such as anxiety- and depression-like behaviors, motor function, social behavior, and learning and memory^23^. Middle aged-animals (8–12 months old) showed more pronounced changes in most of these behaviors than at younger ages (2–6 months old). In this study, we sought to further investigate whether MS affects histone modification and DNA methylation at the p11 promoter from young adult (2 months old) to middle ages (8 months old).

## Results

### Effects of MS on immobility in the FST and hippocampal p11 expression in young adult and middle-aged mice

**Figure 1:**
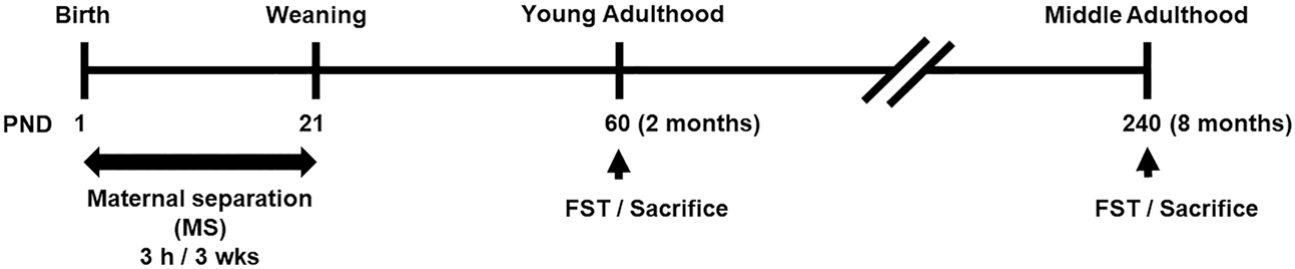
Experimental design timeline. Pups were separated from their mothers for 3 h daily from postnatal day (PND) 1 to PND 21. When the pups reached young adulthood (2 months) or middle age (8 months), they were subjected to the forced swimming test (FST). Following the FST, the mice were sacrificed immediately for the p11 molecular analysis. Molecular analysis was measured within the same batch experiment in all groups.

**Figure 2:**
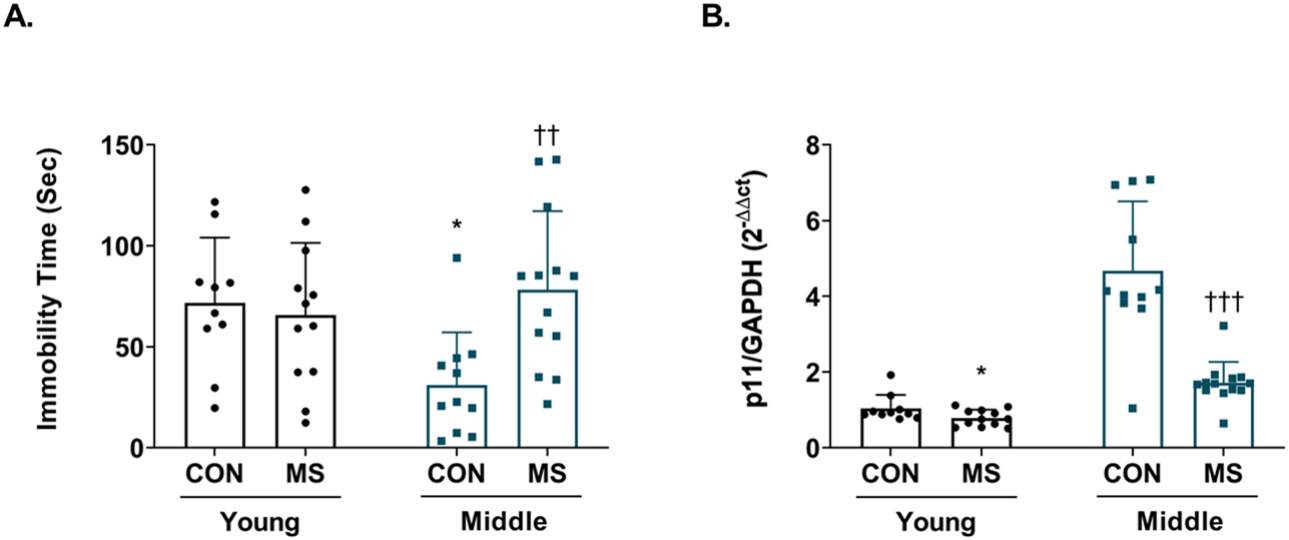
Changes in immobility on the FST and hippocampal p11 levels in young adult and middle-aged mice following maternal separation (MS) exposure. (**A**) Immobility time in the FST was measured at 2 months (young adulthood) or 8 months (middle adulthood). (**B**) p11 mRNA levels in the hippocampus were measured using quantitative real-time polymerase chain reaction (qRT-PCR). All analyses were performed on the hippocampus. All quantities were normalized to glyceraldehyde-3-phosphate dehydrogenase (GAPDH). Data are expressed as values relative to mean ΔCt (control group on young adulthood) using the 2^−ΔΔCt^ method and are presented as mean ± standard error of the mean (SEM; *n* = 10–12/group, young adulthood; *n* = 11–13/group, middle adulthood) ^*^p < 0.05 vs. control in young adulthood; ^††^*p* < 0.01 vs. control in middle adulthood; ^†††^*p* < 0.001 vs. control in middle adulthood.

Young adult and middle-aged MS animals and control animals were tested on FST according to the experimental design timeline (Fig. 1). In two-way ANOVA analysis (Table 1), age effect did not influence immobility time (*F*_(1,42)_ = 1.941, *p* = 0.171). However, we observed the significance on MS effect (*F*_(1,42)_ = 4.172, *p* = 0.047) and interaction effect between age and MS (*F*_(1,42)_ = 6.940, *p* = 0.012). In post hoc test, control and MS animals in young adulthood did not significantly differ in immobility time (*p* = 0.976). Conversely, middle aged-MS animals were significantly more immobile than controls (control = 31.03 ± 7.86 s, MS = 78.18 ± 10.82 s; *p* = 0.008; Fig. 2A). Post hoc test showed a significantly lower immobility time in middle aged control animals compared to young adult controls (young control = 71.63 ± 10.26 s., middle control = 31.03 ± 7.86 s, *p* = 0.044).

**Table 1:** Summary of statistical analysis by two-way ANOVA.

	Main effects				Interaction	
	Age effect		MS effect		Age × MS effect	
	*F*_*(1,42)*_	*p*	*F*_*(1,42)*_	*p*	*F*_*(1,42)*_	*p*
FST	1.941	0.171	4.172	**0.047**	6.940	**0.012**
p11	63.950	** < 0.001**	31.780	** < 0.001**	22.520	** < 0.001**
AcH3	33.500	** < 0.001**	52.420	** < 0.001**	3.663	0.062
H3K4me3	129.000	** < 0.001**	26.660	** < 0.001**	5.153	**0.028**
H3K27me3	21.640	** < 0.001**	20.330	** < 0.001**	2.155	0.150
MSP	3.513	0.068	4.142	**0.048**	4.555	**0.039**

Bold values denote statistical significance at the *p* < 0.05 level.

Ac-H3: histone H3 acetylation; ANOVA: analysis of variance; FST: forced swimming test; H3K4me3: histone H3 trimethylation at lysine residue 4; H3K27me3: histone H3 trimethylation at lysine residue 27; MS: maternal separation; MSP: methylation-specific polymerase chain reaction.

Hippocampal p11 mRNA expression levels between control and MS animals in young and middle adulthood were assessed. Two-way ANOVA revealed that both age (*F*_(1,42)_ = 63,950, *p* < 0.001) and MS (*F*_(1,42)_ = 31.780, *p* < 0.001) affected p11 mRNA levels (Table 1). Moreover, the statistical analysis showed the interaction of these factors (*F*_(1,42)_ = 22.520, *p* < 0.001). In post hoc comparison, MS animals showed a significant reduction in p11 mRNA in young (control = 1.04 ± 0.11, MS = 0.79 ± 0.06; *p* = 0.049) and middle (control = 4.68 ± 0.55, MS = 1.71 ± 0.02; *p* < 0.001) adulthood (Fig. 2B).

### Effects of MS on histone acetylation and methylation at the hippocampal p11 promoter of young adult and middle-aged mice

**Figure 3:**
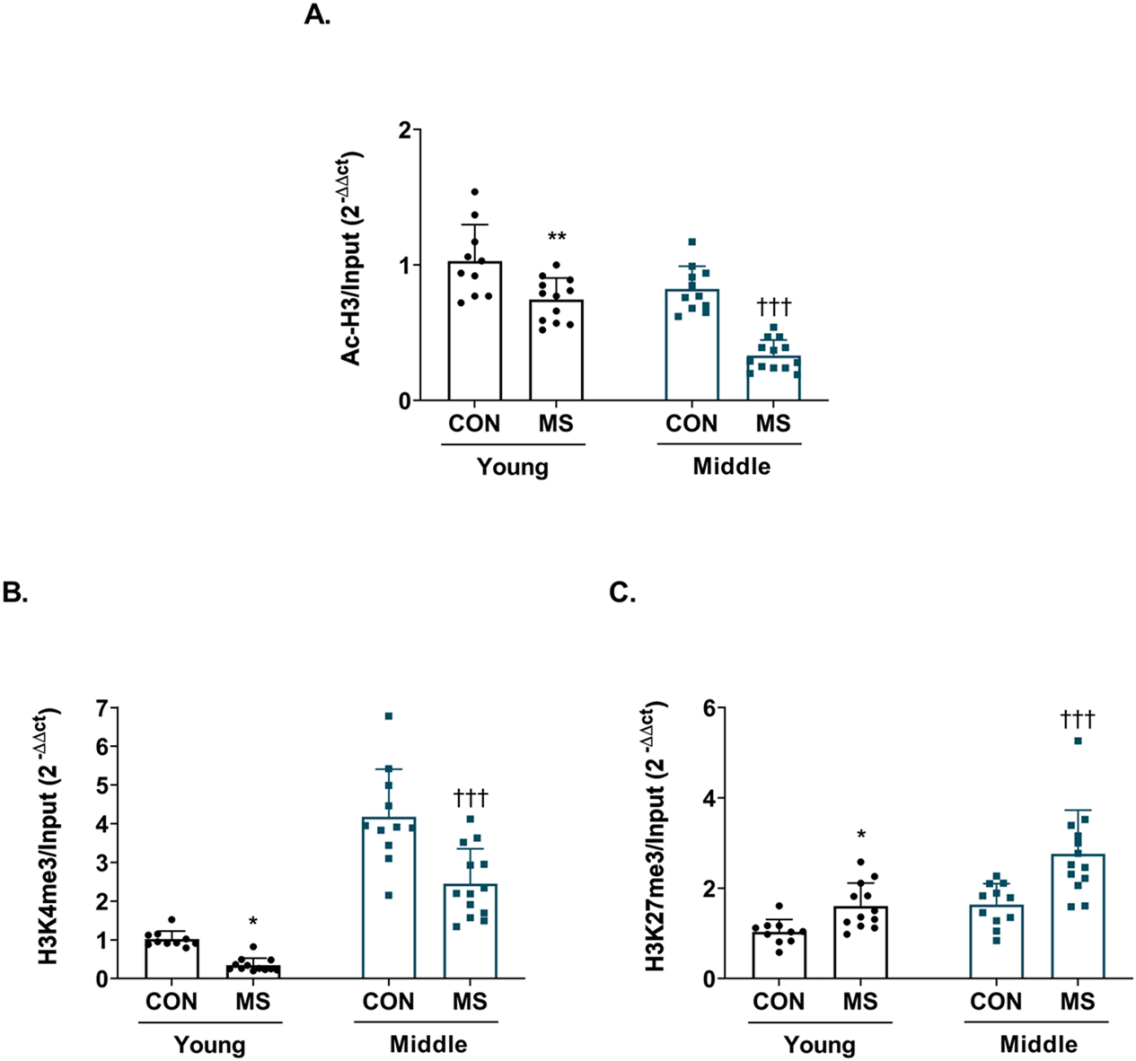
Alterations in histone acetylation and methylation of the hippocampal p11 promoter in young adult and middle-aged mice following MS exposure. The levels of histone H3 acetylation (AcH3, **A**), histone H3 trimethylation at lysine residue 4 (H3K4me3, **B**), and histone H3 trimethylation at lysine residue 27 (H3K27me3, **C**) at the p11 promoter in the hippocampus were measured using a chromatin immunoprecipitation assay with antibodies to AcH3, H3K4me3, and H3K27me3. All analyses were performed on the hippocampus. Data were normalized to input DNA and are expressed as values relative mean ΔCt (control group on young adulthood) using the 2^−ΔΔCt^. The data are presented as mean ± SEM (*n* = 10–12/group, young adulthood; *n* = 11–13/group; middle adulthood). ^*^p < 0.05 vs. control in young adulthood; **p < 0.01 vs. control in young adulthood; ***p < 0.001 vs. control in young adulthood; ^†††^*p* < 0.001 vs. control in middle adulthood.

Epigenetic histone modifications at the p11 promoter were examined in control and MS mice in young and middle adulthood. In two-way ANOVA, both age (*F*_(1,42)_ = 33.500, *p* < 0.001) and MS (*F*_(1,42)_ = 52.420, *p* < 0.001) significantly influenced acetylated histone levels (Table 1). There was a trend for the interaction between age and MS (*F*_(1,42)_ = 3.663, *p* = 0.062) Post hoc test showed that histone acetylation at the hippocampal p11 promoter was reduced in both young adult (control = 1.03 ± 0.09, MS = 0.74 ± 0.05; *p* = 0.003) and middle-aged (control = 0.83 ± 0.01, MS = 0.33 ± 0.03; *p* < 0.001) MS mice compared to matched controls (Fig. 3A).

The level of H3K4 trimethylation, a marker of histone modification activation, was affected by both age (*F*_(1,42)_ = 129.000, *p* < 0.001) and MS (*F*_(1,42)_ = 26.660, *p* < 0.001) as well as their interaction (*F*_(1,42)_ = 5.153, *p* = 0.028; Table 1). Post hoc test confirmed that H3K4 trimethylation of the p11 promoter was reduced in MS mice of young (control = 1.02 ± 0.07, MS = 0.35 ± 0.05; *p* = 0.043) and middle adulthood (control = 4.17 ± 0.37, MS = 2.45 ± 0.25; *p* < 0.001) compared to matched controls (Fig. 3B).

The level of H3K27 trimethylation, a marker of histone modification repression, was significantly affected by both MS (*F*_(1,42)_ = 21,640 *p* < 0.001) and MS (*F*_(1,42)_ = 20.330, *p* < 0.001), but not an interaction (*F*_(1,42)_ = 2.155, *p* = 0.150; Table 1). In post hoc comparison, H3K27 trimethylation was increased greatly in MS mice of both young (control = 1.03 ± 0.09, MS = 1.61 ± 0.15; *p* = 0.047) and middle adulthood (control = 1.63 ± 0.14, MS = 2.76 ± 0.27; *p* < 0.001; Fig. 3C).

### Effects of MS on DNA methylation at the hippocampal p11 promoter of young adult and middle-aged mice

**Figure 4:**
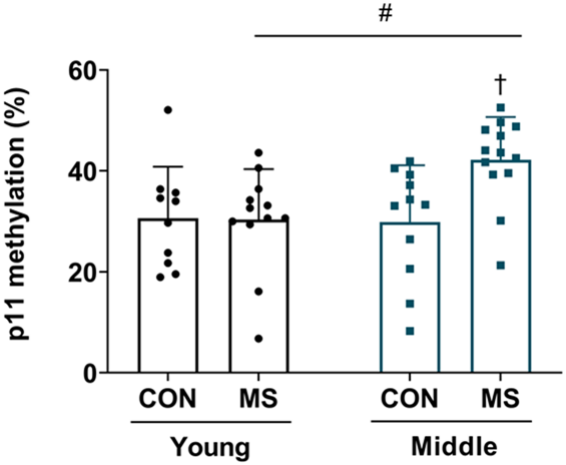
Alterations in DNA methylation at the hippocampal p11 promoter in young adult and middle-aged mice following MS exposure. DNA methylation levels at the hippocampal p11 promoter were measured using methylation-specific polymerase chain reaction (MSP). All analyses were performed on the hippocampus. Ct values were normalized to GAPDH. Data are expressed as percentage of methylated DNA described in “Methods” and are represented as mean ± SEM (*n* = 10–12/group, young adulthood; *n* = 11–13/group, middle adulthood). ^†^*p* < 0.05 vs. control in middle adulthood; ^#^*p* < 0.05 vs. MS in young adulthood.

CpG methylation at the p11 promoter was examined in the hippocampus of young adult and middle-aged MS and control animals. The level of DNA methylation was affected by MS (*F*_(1,42)_ = 4.142, *p* = 0.048). Age did not affect this level (*F*_(1,42)_ = 3.513, *p* = 0.068), however, there was an effect of age and MS interaction (*F*_(1,42)_ = 4.555, *p* = 0.039, Table 1).While DNA methylation at the p11 promoter did not differ between MS and control mice in young adulthood (*p* > 0.999), it was significantly increased in the middle-aged MS animals (control = 29.87 ± 11.00%, MS = 42.18 ± 13.00%; *p* = 0.022; Fig. 4). Moreover, middle-aged MS animals had higher level than young adult MS animals (*p* = 0.025).

## Discussion

This paper reports that MS in early life exerts negative effects on epigenetic mechanisms associated with decreased p11 expression in adulthood, and these effects become more pronounced with age.

In present study, MS had no effect on immobility in young adult mice, but increased immobility was observed in middle age. In a past study, young adult (2 months) MS mice did not affect immobility in the FST; however, MS mice in middle adulthood (8 months) showed increased immobility time^13^, a finding replicated here. Most MS studies reported increased immobility in the FST in young adult animals^24^, although some papers are inconsistent. The findings seem to depend on the strain of mice used in the study. Similar to the MS model used here, Ruiz et al. found that MS (3 h daily from PND 1 to PND 14) in rats increased immobility time at two ages; furthermore, middle-aged (10 months) rats had longer immobility times than young adult (4 months) rats^25^. Taken together, early MS significantly increased immobility in middle adulthood than in young adulthood. The present study showed that immobility time was reduced in middle aged-control animals compared to young adult controls. Similar result has been reported in young age (2 months) and middle aged (8 months)-controls of male C57BL6J mice^13^. In large-scale analysis of the FST and tail suspension test in male C57BL6J mice, immobility times in the 8–12 months old group (*n* = 70) were lower than those in the 4–5 months (*n* = 357) and 2–3 months (n = 495) old groups^23^. Overall, the results of tests in C57BL6J mice indicate that immobility decreases with age.

In this study, MS is associated with a reduction of hippocampal p11 expression at two time points in adulthood. Previous studies have demonstrated that chronic stress in adults induces p11 loss, as well as depression-like behaviors^26,27^. This study is the first to show an association between ELS and p11 levels. In mice, p11 overexpression increases 5-HT_1B_ receptor function and is associated with reduced immobility in the tail suspension test^15^. In contrast, in p11 knockout mice, the number of 5-HT_1B_ receptors is reduced at the cell membrane^15^. Notably, MS (3 h daily from PDN 2 to PND 13) in a rat ELS model reduced hippocampal 5-HT_1B_ receptor binding when assayed through [^125^I] cyanopindolol autoradiography^28^. The reduced binding may result from ELS-induced reductions of p11 levels.

Reduced hippocampal p11 expression in young adult and middle-aged MS animals was associated with altered histone modifications (i.e., significant decreases in H3 acetylation and H3K4me3 and a significant increase in H3K27me3) at the p11 promoter region. Moreover, p11 expression, H3 acetylation, and H3K4 trimethylation after MS were more severely perturbed in middle adulthood. Suri et al. demonstrated that MS induces opposing age-dependent effects on the expression of histone modifying enzymes such as histone deacetylase (*Hdac1* to *Hdac11*) and histone methyltransferase (*G9a* and *Suv391*) in young adulthood (2 months) versus middle adulthood^29^. Nonetheless, the altered expression of these enzymes did not affect overall H3 acetylation, H3K9me2, and H3K9me3 at either of the ages. Although histone modifying enzymes may not globally change histone modification across the lifespan, these enzymes may differentially regulate histone modifications at the promoters of stress-related genes during aging. A loss of balance between epigenetic modifications during aging is referred to as “epigenetic drift”^30^. The global distribution of many histone acetylation and methylation modifications (i.e., H3K27me3, H3K56ac, and H4K16ac) has been found to be altered during aging in various organisms^31,32^. Accordingly, MS animals in middle age, but not young adulthood, show histone modifications associated with decreased BDNF IV expression concurrent with impairments in hippocampal-dependent cognition^10^. A similar age-dependent effect on histone acetylation and methylation at the GR exon I_7_ promoter has been observed^13^. Taken together, middle-aged MS animals may exhibit more severe changes in histone modification than young adult MS animals due to age-related epigenetic drift.

Increased DNA methylation in p11 promoter regions following MS were only present in middle-aged mice, not those in young adulthood. The increased DNA methylation, along with the altered histone modifications, could account for the reduced p11 expression in middle-aged MS animals. In accordance, a negative correlation between p11 gene transcription and CpG DNA methylation at the p11 promoter has been reported^33^. Moreover, reduced p11 levels in a genetic rodent model of depression were associated with higher DNA methylation, while escitalopram treatment elevated p11 levels and reduced DNA methylation of the p11 promoter.

**Figure 5:**
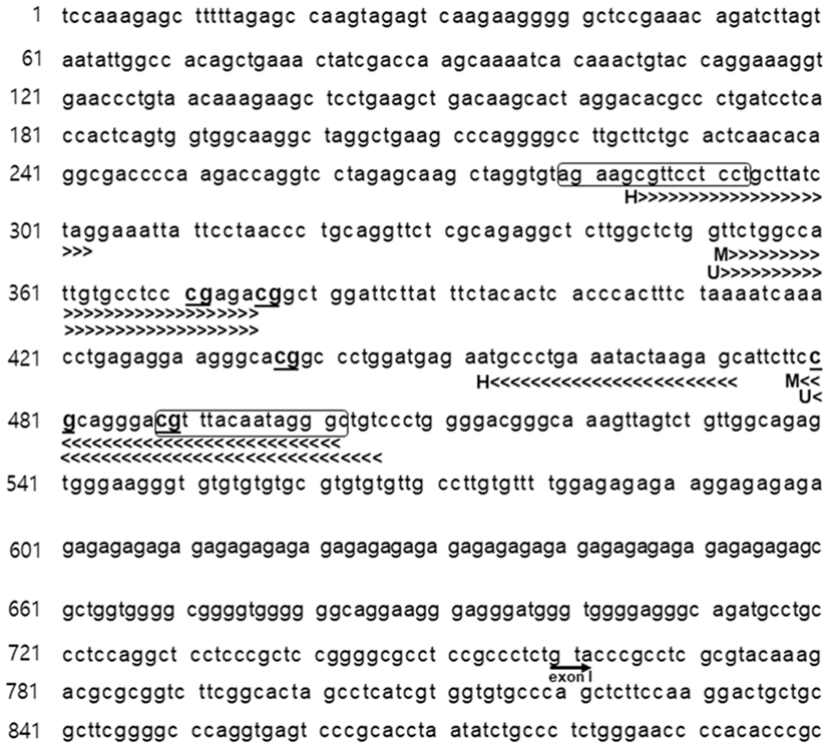
p11 proximal DNA sequence. This sequence represents the promoter region (bases 1–660) and exon I (bases 760 ~) sequence, corresponding to bases 93,554,358–93,555,257 of the NCBI reference sequence NC_000069.6 (latest) investigated. H>>>>> and H<<<< are forward (bases 284–303) and reverse (bases 453–472) primer regions, respectively, for histone modifications of the p11 promoter. M>>>> and M<<<< are forward methylation-specific (bases 353–376) and reverse methylation-specific (bases 479–502) primer regions, respectively, for DNA methylation of the p11 promoter. U>>>> and U<<<< are forward unmethylation-specific (bases 352–376) and reverse unmethylation-specific (bases 480–506) primer regions, respectively, for DNA methylation of the p11 promoter. The first encircled region is the putative androgen receptor binding site, and the second encircled region is the putative glucocorticoid receptor binding site. CpG sites investigated in this study are underlined and bolded.

In rodents, the absence of maternal care such as licking, grooming, and arched-back nursing, is associated with hypothalamic–pituitary–adrenal (HPA) axis dysregulation as identified by increased glucocorticoid levels^34^. The lack of maternal care modifies DNA methylation, resulting in decreased hippocampal GR expression^35^. Accordingly, adults with histories of childhood maltreatment show increased DNA methylation of the GR exon I_F_ promoter and, consequently, increased HPA activity^36,37^. GR acts as a ligand-activated transcription factor. When activated by glucocorticoids, GR translocates to the nucleus and binds to GR binding sites within the promoters of target genes, thereby activating or repressing their expression^38^. p11 is a target gene for GRs. Putative GR binding sites were identified using the web-based tools described in “Methods” section; this site includes one CpG site (Fig. 5). Zhang et al. reported that GR increases p11 promoter activity via the interaction of glucocorticoid-bound GR with GR binding sites^39^. Thus, elevated GR levels may activate p11 transcription. Hippocampal GR expression levels have been found to be reduced in young adult and middle-aged MS animals and, furthermore, the reduction in middle adulthood was remarkably higher than in young adulthood^13^. Thus, the differential reduction in p11 levels in middle-aged MS animals could be due to CpG methylation of the GR binding site, which prevents GR from easily accessing the p11 promoter, resulting in reduced p11 transcriptional activity. Nevertheless, further experiments are needed to address whether MS causes an age-dependent decrease in nuclear GR levels.

Research on the long-term effects of ELS suggests that ELS can be beneficial or detrimental depending on factors such as social support, enriched environment, and additional stress. Clinical study has shown that subjects with ELS experience reduced their vulnerability to stress by reducing stress and negative effects in daily life though work-related social support^40^. In maternally separated rats, exposure to an enriched environment during adolescence enhanced cognitive function and BDNF signaling in the hippocampus^41^. On the other hand, the combination of early-life MS and adult social defeat stress reveled significant dysregulation of the histone methylation, BDNF, β-catenin, and GR signaling, indicating that ELS increases vulnerability to stress^42^.

There are several limitations to the present study. Firstly, we performed FST to assess the depression-like behavior. It is not sufficient to use only FST. Additional behavioral tests are needed, such as the tail suspension test to measure behavioral despair state or the sucrose preference test to measure anhedonia. Also, behavioral tests that measure reward and arousal may be more appropriate. In particular, the effects of ELS on learning and memory are well established, and there is evidence that these effects worsen with age^43^. The Morris water maze test, which measures hippocampal dependent learning and memory, may also be appropriate. Secondly, the weakness of the current study is that it has only been tested in male mice. Epidemiological studies have shown that depression is more prevalent in women than men^44^. For depression-related studies, some researchers preferred male mice to female ones due to variability in the estrus cycles^45–50^. Others, on the other hand, used both male and female animals during their studies, and the baseline variation in females was not greater than that of males^51–54^. In further study, it is desirable to investigate both male and female mice. Finally, we did not control the effects of litter, but it’s important to take this into account. Variation between dams/litters can affect behavioral and molecular outcomes. Therefore, if more than one animal is used from each litter, they should be averaged to an *n* = 1 to control for litter effects. The effect of MS on the immobility of FST is also age dependent. However, we cannot conclude that the results of FST in the current study reflect only epigenetic modification of the p11 gene. This is because epigenetic mechanisms of other stress-related genes may also be involved. Changes in p11 expression and epigenetic regulation with age may partially mediate the behavioral change associated with the state of despair seen in FST. In conclusion, this study demonstrates that MS has age-dependent effects on the expression of p11 gene that is associated with changes in histone modification and DNA methylation. Furthermore, the deleterious effects are more severe with age.

## Methods

### Animals

The animal experiments were approved by the Institutional Animal Care and Use Committee (IACUC) at the College of Medicine Inje University (approval no. 2016-053) and performed in accordance with the IACUC and the ARRIVE^55^ guidelines. All animals were anesthetized with carbon dioxide (CO_2_) according to the American Veterinary Medical Association (AVMA) Guidelines for the Euthanasia of Animals (2020 Edition). Pregnant C57BL/6J mice (Daehan Biolink, Eumseong, Chungbuk, Korea) arrived at the Inje Medical College animal facility on gestation day 15. A total of 16 pregnant mice were used for this study. Each dam and its litter were housed in standard cages under standard laboratory conditions (21 °C, 12 h/12 h light/dark cycle, food and water available ad libitum).

### Maternal separation (MS)

Each dam and its litters were kept together in each cage. Sixteen dams/their litters were divided randomly into control and MS groups. Pups in the MS group were separated from their mothers for 3 h daily starting on postnatal day (PND) 1 to PND 21 as described previously^56^. Pups in the control group were left undisturbed except during routine animal facility handling. Litters were kept with their dams until PND 35, then separated by sex and randomly housed to 5–7 animals per cage. Only male mice were used. Male control and MS mice were assayed at either 2 months old (young adulthood) or at 8 months old (middle adulthood; Fig. 1). These ages were defined according to the comparison between human and C57BL/6J mouse ages: young adult (2–6 months old) and middle age (8–14 months old)^23,57^..

### Forced swimming test (FST)

The FST was performed in control and MS mice at either young (*n* = 10–12/group) or middle (*n* = 11–13/group) adulthood as described previously^56^. Mice were individually placed in transparent plastic cylinders (25 cm height × 10 cm diameter) containing 12 cm of water (23–25 °C) for 7 min and recorded on video. After an initial 2-min habituation period, the time spent immobile during the remaining 5 min was analyzed.

### Measurement of mRNA levels using quantitative real-time polymerase chain reaction (qRT-PCR)

Following the FST, whole brains were extracted (*n* = 10–12/group, young adulthood; *n* = 11–13/group, middle adulthood). The hippocampus was dissected from the brain; RNA isolation, cDNA synthesis, and qRT-PCR were performed on the hippocampal tissue as described previously^56^. Gene-specific primers (Table 2) for p11 and glyceraldehyde-3-phosphate dehydrogenase (GAPDH) were used under the following conditions: 95 °C for 10 min, followed by 40 cycles of 95 °C for 15 s, 55 °C for 35 s, and 72 °C for 35 s. The cycle threshold (Ct) values were calculated automatically. Ct values were normalized to GAPDH. Quantification was performed using the 2^−ΔΔCt^ method: ΔCt = Ct (target gene) − Ct (GAPDH) and ΔΔCt = ΔCt (sample) − ΔCt (calibrator). The average ΔCt (control on young adulthood) was used as a calibrator. Relative expression = the 2^−(ΔCt (sample) – average ΔCt (control on young adulthood))^ was calculated for each sample.

**Table 2:** Primers used in the study.

	Primer sequence (5′–3′)
**Quantitative real-time polymerase chain reaction (qRT-PCR) for mRNA**	
p11 mRNA^64^^a^	Forward TGCTCATGGAAAGGGAGTTC
	Reverse CCCCGCCACTATTGATAGAA
GAPDH mRNA^65^^b^	Forward AACAGCAACTCCCATTCTTC
	Reverse TGGTCCAGGGTTTCTTACTC
**qRT-PCR for histone modification (chromatin immunoprecipitation [ChIP] assay)**	
p11 promoter^c^ (189 base-pair [bp]; base 93554641–93554829)	Forward CGTTCCTCCTGCTTATCTAG
	Reverse GCTCTTAGTATTTCAGGGCA
**qRT-PCR for DNA methylation (methylation-specific polymerase chain reaction [MSP] analysis)**	
p11 promoter^c^	
Methylation-specific: (150 bp; base 93554710–93554859)	Forward TTTGGTTATTGTGTTTTTCGAGAC
	Reverse ACCCTATTATAAACGTCCCTACGA
Unmethylation-specific: (155 bp; 93554609–93554863)	Forward TTTTGGTTATTGTGTTTTTTGAGAT
	Reverse AACAACCCTATTATAAACATCCCTACA

^a^*Mus musculus* S100 calcium binding protein A10 (calpactin; S100a10), mRNA; NCBI Reference Sequence: NM_009112.2.

^b^*Mus musculus* glyceraldehyde-3-phosphate dehydrogenase (GAPDH), pesudogene 14 (Gapdh-ps14) on chromosome 8; NCBI Reference Sequence: NG_007829.2.

^c^*Mus musculus* strain C57BL/6J chromosome 3, GRCm38.p4 C57BL/6J; NCBI Reference Sequence: NC_000069.6 (GenBank Assembly ID: GCF_000001635.24).

### Chromatin immunoprecipitation (ChIP) assays

Chromatin was extracted from the isolated hippocampus using a standard protocol (SimpleChIP^®^ Plus Enzymatic Chromatic IP Kit; Cell Signaling, Beverly, MA, USA) as described previously (*n* = 10–12/group, young adulthood; *n* = 11–13/group, middle adulthood)^56^. Primers were designed around a putative p11 promoter region (Table 2 and Fig. 5). Because the p11 promoter is not well characterized in mice, the region of interest was created based on previous data on epigenetic alterations in the rat p11 promoter^33,58^. The putative proximal promoter of the p11 gene (~ 700 base-pair upstream region) includes transcription factor binding sites generated by PROMO^59,60^ and MotifMap^61,62^, two freely available web-based tools that identify presumptive transcription factor binding sites (Fig. 5).

Chromatin was immunoprecipitated with antibodies against histone H3 acetylated at K9 and K14 (AcH3, 06–599; Millipore Sigma, Billerica, MA, USA), histone H3 trimethylated at K4 (H3K4me3, ab8580; Abcam, Cambridge, MA, USA), and histone H3 trimethylated at K27 (H3K27me3, ab6002; Abcam) using a SimpleChIP^®^ Plus Enzymatic Chromatic IP Kit. To confirm antibody specificity, chromatin samples were immunoprecipitated with ChIP antibodies and normal rabbit IgG (#2729; Cell Signaling). qRT-PCR was performed on purified DNA using a control primer set (SimpleChIP^®^ Mouse RPL30 Intron 2 Primers #7015; Cell Signaling) and p11 promoter primers (Figure S1). Ct values were normalized to input DNA. Quantification was performed using the 2^−ΔΔCt^ method: ΔCt = Ct (immunoprecipitation) − Ct (input) and ΔΔCt = ΔCt (sample) − ΔCt (calibrator). The average ΔCt (control on young adulthood) was used as a calibrator. Relative level = the 2^−(ΔCt (sample) – average ΔCt (control on young adulthood))^ was calculated for each sample.

### Methylation-specific polymerase chain reaction (MSP) analysis

Genomic DNA was extracted from the hippocampus using a QIAGEN DNA prep kit (51036; Valencia, CA, USA) and treated with bisulfite using an EpiTect^®^ Bisulfite kit (59104; QIAGEN). To determine the DNA methylation status of CpG in the p11 promoter region, a qRT-PCR was performed on the same amount of bisulfite-treated DNA using an EpiScope^®^ MSP kit (#R100A; TaKaRa, Otsu, Japan) containing SYBR green (TaKaRa). Specific primers for methylated or unmethylated p11 promoters were designed using MethPrimer^63^. Primers include either a 150 base-pair (methylation-specific) or 155 base-pair (unmethylation-specific) region with 5 CpGs sites in the p11 promoter region (Fig. 5). Primer sequences are listed in Table 2. The p11 promoter region was confirmed to be amplified with methylation- and unmethylation-specific primers (Figure S2). The qPCR reaction conditions were as follows: initial denaturation at 95 °C for 30 s, followed by 40 cycles of denaturation at 98 °C for 5 s, annealing at 53 °C for 30 s, and extension at 72 °C for 1 min. Ct values were normalized to GAPDH, and differences in methylation and unmethylation between control and MS groups (*n* = 10–12/group, young adulthood; *n* = 11–13/group, middle adulthood) were calculated using the ΔCt. Levels of methylated DNA (%) were calculated according to the following formula: methylated rate (%) = ΔCt _methylated DNA_/(ΔCt _methylated DNA_ + ΔCt _unmethylated DNA_) × 100.

### Statistical analysis

GraphPad Prim 8.0 (La Jolla, USA) was used for statistical analysis. To determine the main and interaction effects of age and MS, two-way ANOVA was performed. Turkey’s multiple-comparison tests were used for post hoc comparisons. *P*-values < 0.05 were considered to indicate statistical significance, and all data are presented as means ± standard error of the mean (SEM).

## Supplementary Information

Supplementary Information.

## Data Availability

The data are availability from the corresponding author upon request.
