# Supplementary material for: Early life stress induces age-dependent epigenetic changes in p11 gene expression in male mice
Source: Sci Rep. 2021 Sep 1;11:10663. doi: 10.1038/s41598-021-89593-7 (PMC8410943; doi:10.1038/s41598-021-89593-7)
Supplement: Supplementary file 1 — Supplementary Information. [file 41598_2021_89593_MOESM1_ESM.docx]

**Supplementary Information**

**Early life stress induces age-dependent epigenetic changes in p11 gene expression in male mice**

Mi Kyoung Seo ^1^, Jung Goo Lee ^1,2*^, Sung Woo Park ^1,3^*

^1^ Paik Institute for Clinical Research, Inje University, Busan, 47392, Republic of Korea.

^2^ Department of Psychiatry, College of Medicine, Haeundae Paik Hospital, Inje University, Busan, 48108, Republic of Korea.

^3^ Department of Convergence Biomedical Science, College of Medicine, Inje University, Busan, 47392, Republic of Korea.

* **Corresponding authors**

Sung Woo Park, Ph.D. and Jung Goo Lee, M.D., Ph.D.

Paik Institute for Clinical Research, Inje University, 75 Bokji-ro, Busanjin-gu, Busan, Republic of Korea. Zip code:47392; Tel: +82 51 890 6749; Fax: +82 51 894 6709; E-mail address: swpark@inje.ac.kr (S.-W. Park)/ iybihwc@inje.ac.kr (J.-G. Lee)


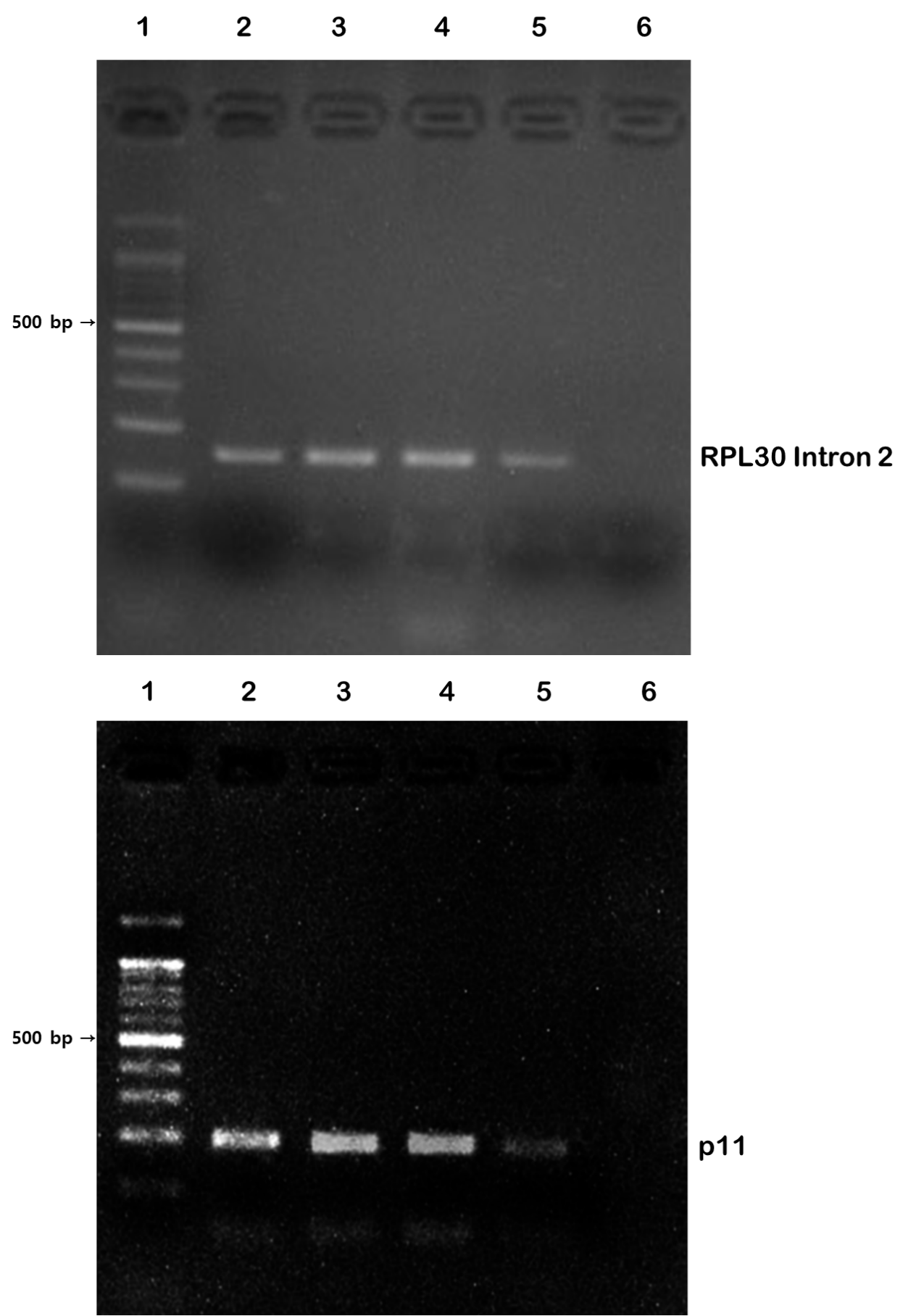


**Figure S1. Antibody specificity in the chromatin immunoprecipitation (ChIP) assay.**

ChIP was performed using digested chromatin from mouse hippocampal tissue and the indicated antibodies. Quantitative real-time polymerase chain reaction (qRT-PCR) was used to analyze purified DNA with the SimpleChIP Mouse RPL30 Intron 2 Promoter Primers #7015 (control primer set; upper bands, 159 base-pairs) and p11 promoter primers (lower bands, 189 base-pairs). qRT-PCR products were observed for each primer set in the input sample (lane 2) and various ChIP samples (lanes 3–5) but not in the normal rabbit IgG ChIP sample (lane 6). PCR products were confirmed by electrophoresis with a 2% agarose gel.

Lane 1: Size marker (100 base-pair ladder, EBM-1001; ELPISBIO)

Lane 2: Positive control; Input sample

Lane 3: AcH3 (K9/K14; 06-599; MILLIPORE)

Lane 4: H3K4me3 (ab8580; ABCAM)

Lane 5: H3K27me3 (ab6002; ABCAM)

Lane 6: Negative control; Normal rabbit IgG (#2729; CELL SIGNALING)


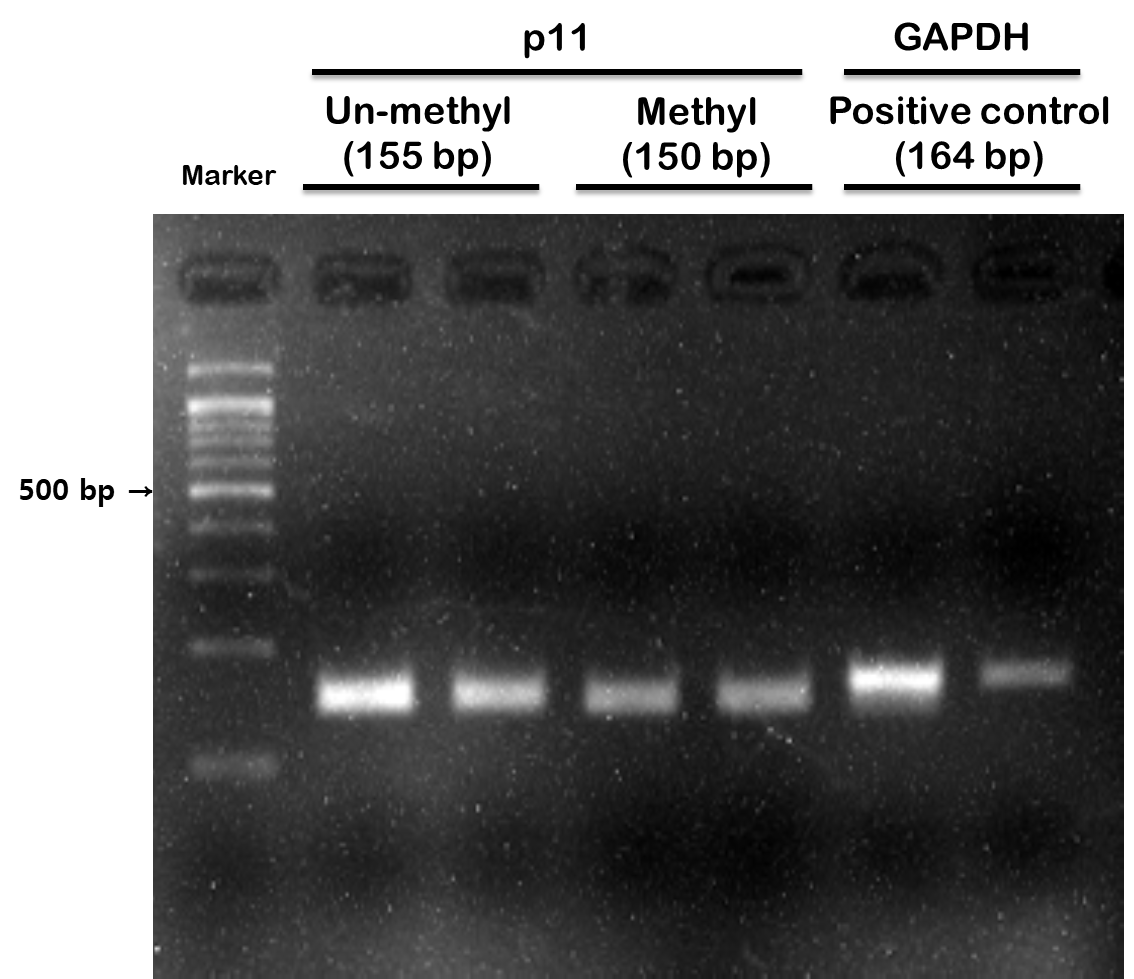


**Figure S2. Polymerase chain reaction (PCR) amplified products using the methylation- and unmethylation-specific primers of the p11 promoter.**

Genomic DNA isolated from hippocampal tissue was bisulfite-converted. Bisulfite-treated DNA was analyzed by PCR using methylation- and unmethylation-specific primers of the p11 promoter. PCR products were confirmed by electrophoresis with a 2% agarose gel.
